# Commentary on substance use disorders and risk of suicide in a general US population: a case control study by Lynch et al.

**DOI:** 10.1186/s13722-020-00195-6

**Published:** 2020-06-29

**Authors:** Virna Little, Mary Crawford James

**Affiliations:** 1 Center for Innovation in Mental Health, City University of New York School of Public Health, 55 W 125th Street, New York, NY 10027 USA; 2 Concert Health, 550 W B St 4th floor, San Diego, CA 92101 USA

**Keywords:** Suicide, Suicide prevention, Suicide screening, Substance use disorders, Emergency departments, Primary care, Integration

## Abstract

In response to the findings of the Lynch et al. study on the association of suicide mortality and substance use disorder, this commentary addresses the need for increased suicide assessment and screening for patients who identify with substance use disorders in health settings such as primary care practices and emergency departments. Lynch et al. found that all categories of substance use disorders were associated with increased risk of suicide. We present the Suicide Safer Care project as one model to address this need. The Suicide Safer Care project contains a training model that provides education on basic principles of suicide prevention and skills for integration into practice. Data collected from pre- and post-training surveys of participants demonstrates the success of the training in increasing knowledge and confidence in identification and treatment of patients at risk of suicide. Utilizing training techniques such as Suicide Safer Care and combining it with the findings from Lynch et al. providers in primary care settings and emergency departments can optimize the number of patients they identify with suicide risk, impacting the number of patients who receive treatment and the number of potential lives saved.

Lynch et al. report findings on the association of substance use disorders (SUDs) and suicide mortality in a general US population. This study found that all categories of SUDs were associated with increased risk of suicide. While most studies on substance use disorders and suicide mortality have been completed among veteran populations, this study of the general US population raises an important issue that should be considered for health systems who may want to increase suicide screening and prevention efforts for patients identified with SUDs, particularly in settings such as primary care or emergency departments.

## Background

Suicide mortality has steadily increased over the last decade, becoming one of the top ten leading causes of death in the US with over 47,000 deaths per year and another 1.4 million estimated attempts [1]. In 2017, an estimated 20.7 million people needed treatment for a substance use disorder, yet only 4 million people received treatment [2]. Due to barriers such as stigma, lack of access to mental health care due to geographic location or cost, and/or lack of mental health diagnosis, many individuals do not seek help for their substance use or suicidality. In turn, many individuals at risk may only have the opportunity to receive care for their behavioral and mental health needs in primary care and emergency settings.

In order to address these barriers, there has been an increase in the utilization of integrated care approaches to address behavioral health issues in physical health settings. This integration increases access to needed care for individuals who may be at risk for suicide or substance use. Primary care and emergency settings have long been recognized as a critical access point to health care systems, and in the past few decades they have been recognized more frequently as an opportunity to identify and link patients to care for suicide and substance use disorders.

On the other hand, research shows that 84% of individuals who die by suicide have a healthcare visit in the year prior to their death, and 45% of individuals who die by suicide visited their primary care provider within the month prior to their death [3]. Additionally, almost 40% of individuals who died by suicide visited the emergency department in the year prior to their death [4]. This places primary care providers (PCPs) and emergency department staff in a unique position to engage patients in conversations around mental health, suicidal ideation, and substance use.

Presently there are broad scale efforts to encourage PCPs and providers in other settings to expand depression screening, including suicide risk. This is often a short process where patients are asked to answer two questions through the PHQ2 screening, rather than initially completing the longer nine-question screening that includes questions directly about suicide. Subsequently, general medical settings are being encouraged to practice Screening, Brief Intervention, and Treatment (SBIT), which includes screening for substance use or misuse, as well as provision of medications for opioid use disorder (MOUD) [5]. There is little focus on developing processes in which these screening efforts are optimized or personalized. Helping providers understand the research and correlation between substance use and suicide will encourage the development of screening protocols and algorithms that automatically include specific questions about suicide for patients who identify with substance use disorders.

The Suicide Safer Care training project was created as a means to increase screening, assessment, and treatment of patients at risk of suicide in multiple settings, including primary care and emergency departments [6, 7]. Through a brief, 90 min training, all staff (from CEO, to management, to technicians, etc.) within a practice are trained on basic principles of suicide prevention and skills for integration into practice. The training provides a comprehensive, skills-based learning opportunity that offers hands-on strategies that can be used with patients during a healthcare visit. Skills developed during the training include identification of patients at risk, conducting risk assessments using a standardized tool, and brief evidence-based interventions including strategies for reducing access to lethal means and safety planning.

An analysis of surveys conducted with Suicide Safer Care participants prior to the start of training found that 91% of the participants believed that suicide prevention was an important part of their role, including 93% of primary care providers and 89% of health care team members. However, one-third of health care team members and primary care providers had not received training on how to recognize the warning signs that a patient may be at elevated risk for suicide. Survey responses from providers prior to attending the training highlighted the importance of addressing suicide care in primary care, such as:

- 36% of primary care providers had interacted at least once with a patient who ended his or her life by suicide.
- 57% of primary care provider did not feel confident in their ability to provide treatment to patients with suicidal thoughts or behaviors.

The Suicide Safer Care training has been delivered to over 2000 individuals across eight states in the past year and a half. In addition to the in-person live trainings, web-based trainings are conducted to expand the reach of the educational offerings. Data collected in pre- and post-training surveys indicates the success of the program in bridging the knowledge gap in health care professionals’ treatment of patients with suicide ideation. One important finding of the project has been the tremendous response from providers and other care team members in the interest and desire to receive training on how to assess patients at risk and care for patients that are identified with elevated risk for suicide, such as those identified with substance use disorders.

SBIT is an evidence-based intervention that is used to identify at-risk substance users in non-SUD treatment settings, such as general medical settings, while Suicide Safer Care seeks to identify individuals at risk for suicide in a similar fashion. The ideal scenario would be universal screening of all patients for both suicide risk and substance use disorders, but with the correlation between substance use and suicide risk, providers can at the very least incorporate components of Suicide Safer Care into SBIT (Table 1).

**Table 1:** Recommended components of Suicide Safer Care for SBIT

Identification
Patient Health Questionnaire (PHQ-9): Evidence-based screening tool for depression that can be modified for different populations. Question #9 specifically addresses suicide risk by asking about “thoughts that you would be better off or hurting yourself in some way” PHQ-9 Overview [8]
Assessment
Columbia Suicide Severity Rating Scale (C-SSRS): Evidence-based tool that can be used to identify whether a patient has thought about suicide, taken action or plans to take action, or whether they have attempted suicide or plan to attempt suicide C-SSRS Resource [9]
Ask Suicide Questions (ASQ): The ASQ is a brief questionnaire containing 4 items and can be utilized in all populations and practice settings (ED, inpatient medical, primary care settings) to identify youth at risk of suicide ASQ Toolkit [10]
Intervention
Safety Planning: Created in conjunction with patient and provider (and sometimes family members) to identify triggers and warning signs, specific activities that can steer the patient from suicidal thoughts, contacts to be used during times of distress (community, professional, and emergency), etc., in order to alleviate a suicidal crisis Suicide Safety Planning Guide [11]
Counseling on Access to Lethal Means (CALM): Anyone who is identified with any level of suicide risk, from mild to severe, should be asked about access to lethal means. CALM is a workshop that helps providers identify patients who would benefit from lethal means counseling strategies, ask about access to lethal mans, and work with patients and their family to reduce access CALM Resource [12]

## Conclusions

In conclusion, the Lynch et al. findings, along with the results of the Suicide Safer Care project, demonstrate the crucial need for increased suicide risk screening and assessment among patients at risk for suicide, including those identified with SUDs. This commentary agrees with the call to action from Lynch et al. that health systems need to consider suicide prevention screening for patients who are identified with substance use disorder, and we have presented one model currently being implemented around the country to accomplish this task. This includes education, training, and implementation steps to give providers in all health settings the resources and tools they need to feel confident and knowledgeable in the care they provide for SUDs and suicide. Educating providers on how to utilize their existing screening efforts to optimize the number of patients they identify with suicide risk will greatly impact the number of patients who receive care and treatment, as well as the number of potential lives saved.

## Data Availability

Not applicable.

## Abbreviations

SUDs: Substance use disorders
PCPs: Primary care providers
MOUD: Medications for opioid use disorder
SBIT: Screening, brief intervention and treatment
